# Regulatory T-Cells: Potential Regulator of Tissue Repair and Regeneration

**DOI:** 10.3389/fimmu.2018.00585

**Published:** 2018-03-23

**Authors:** Jiatao Li, Jean Tan, Mikaël M. Martino, Kathy O. Lui

**Affiliations:** ^1^Department of Chemical Pathology, Prince of Wales Hospital, The Chinese University of Hong Kong, Hong Kong, Hong Kong; ^2^Li Ka Shing Institute of Health Sciences, Prince of Wales Hospital, The Chinese University of Hong Kong, Hong Kong, Hong Kong; ^3^European Molecular Biology Laboratory Australia, Australian Regenerative Medicine Institute, Monash University, Melbourne, VIC, Australia

**Keywords:** CD4^+^ regulatory T-cells, tissue repair and regeneration, stem cells, macrophages, heart regeneration

## Abstract

The identification of stem cells and growth factors as well as the development of biomaterials hold great promise for regenerative medicine applications. However, the therapeutic efficacy of regenerative therapies can be greatly influenced by the host immune system, which plays a pivotal role during tissue repair and regeneration. Therefore, understanding how the immune system modulates tissue healing is critical to design efficient regenerative strategies. While the innate immune system is well known to be involved in the tissue healing process, the adaptive immune system has recently emerged as a key player. T-cells, in particular, regulatory T-cells (Treg), have been shown to promote repair and regeneration of various organ systems. In this review, we discuss the mechanisms by which Treg participate in the repair and regeneration of skeletal and heart muscle, skin, lung, bone, and the central nervous system.

## Introduction

The global number of individuals suffering from organ dysfunction as a result of acute injuries, chronic disorders, or aging has been on the rise and thus inadvertently places a high demand for organ transplantation. However, organ and tissue transplantation is obviously limited by the shortage of donors and side effects associated with the use of immunosuppressants (1), placing stress upon current methodology and creating a need for an alternative therapeutic avenue. By virtue of its self-renewal properties and capability in differentiating into multiple cell types, recent advances in human pluripotent stem cell research has offered a literally unlimited amount and varieties of therapeutic cells for transplantation (2, 3). Nevertheless, there is a lack of clinical evidence showing their long-term engraftment following transplantation possibly due to poor cell survival and chronic immune rejection (4, 5). Moreover, regenerative therapies stimulating endogenous regeneration such as growth factor-based strategies have shown mixed results in the clinic due to safety concerns and cost-effectiveness (6, 7). Therefore, it is necessary to find new ways to improve regenerative strategies and one of them is to control and utilize the host immune system. Nevertheless, in order to design immune-centric regenerative therapies, it is imperative to understand how the various immune components modulate tissue repair and regeneration.

Since decades, the immune system is well known to be implied in tissue repair and regeneration. For instance, inflammation following injury greatly contributes to tissue repair and scar formation, while excessive inflammation led by immune cells causes pathological fibrosis that debilitates tissue function and may lead to organ failure. Immune-mediated tissue healing processes are complex, yet, highly orchestrated. After injury, invading pathogens, necrotic debris, the clotting reaction, and tissue-resident immune cells trigger an inflammatory response, which result in the recruitment of various immune cells. The activity of immune cells during wound healing can be separated in three phases (8): First, pro-inflammatory cells are recruited to the site of injury for host defense and phagocytosis of necrotic tissues. Second, the pro-inflammatory response is dampened *via* immune cells such as anti-inflammatory macrophages, while immune cells also directly participate in stimulating angiogenesis, myofibroblast activation, and tissue progenitor cell proliferation. Last, most immune cells exit the site of injury or are eliminated by apoptosis and the tissue homeostasis is restored. Nonetheless, the role of the various immune cells and their subsets as well as the mechanisms by which they regulate tissue healing remain largely elusive. It is, therefore, imperative to understand how tissue healing is controlled by the immune system and harnessing the endogenous regenerative capacity has recently become an active area of research.

An interesting observation supporting the critical role of immunity in regeneration (as opposed to tissue repair and scarring) comes from the evolution of the immune system among species and during development. Compared to lower vertebrates such as amphibians and teleost fishes that are capable of completely regenerating many body parts, mammals have a limited regenerative potential. To explain this difference, it has been postulated that the loss of regenerative capacity in mammals is in part associated with maturation of their immune system compared to lower vertebrates (9, 10). The immune system also changes during development and throughout life. For example, some organs such as the mammalian heart is notorious for not being able to regenerate and the necrotic cardiac muscles are replaced by dysfunctional scar tissues after injury. However, accumulating evidence shows that the neonatal hearts of mammals including humans have a transient regenerative capacity compared to adults (11–13). Indeed, the mammalian adaptive immune system is relatively immature after birth, which coincides with the period of neonatal regeneration. In contrast to adults, neonates do not mount a robust fibrotic but a more angiogenic response that facilitates tissue regeneration after injury (10). Therefore, since immune cells regulate both fibrosis and angiogenesis during tissue healing, targeting the immune system to promote neoangiogenesis with minimal fibrosis would be an interesting approach to stimulate regeneration. Therefore, understanding how immunity regulates tissue fibrosis and neoangiogenesis would shed light on the development of potential therapeutics targeting endogenous tissue regeneration. During the last decade, innate immunity, in particular, macrophages and their various polarization states, has been considered as a central regulator of the tissue healing process. However, recent evidences suggest that the adaptive immune system is also a critical actor. In this review, we focus on the role of regulatory T-cells (Treg).

## Overview of the Immune Functions of Treg During Tissue Healing

Treg are required for maintenance of self-tolerance, preventing excessive inflammation and autoimmune diseases. The most reliable cell-specific marker of Treg is Forkhead box P3 (FOXP3), which is essential for the maturation and function of Treg. Congenital deficiency in Treg, due to mutation of the *Foxp3* gene, causes fatal autoimmunity in mice, the scurfy phenotype, and enteropathy, X-linked (IPEX) syndrome in human (14, 15). Treg are normally present in lymphoid organs but have been shown to accumulate in damaged tissues to some extent. Long recognized as potent suppressors of the immune system, Treg have been recently rediscovered as indirect and direct regulators of tissue healing, while the mechanisms are still largely unknown (16–18).

**Figure 1:**
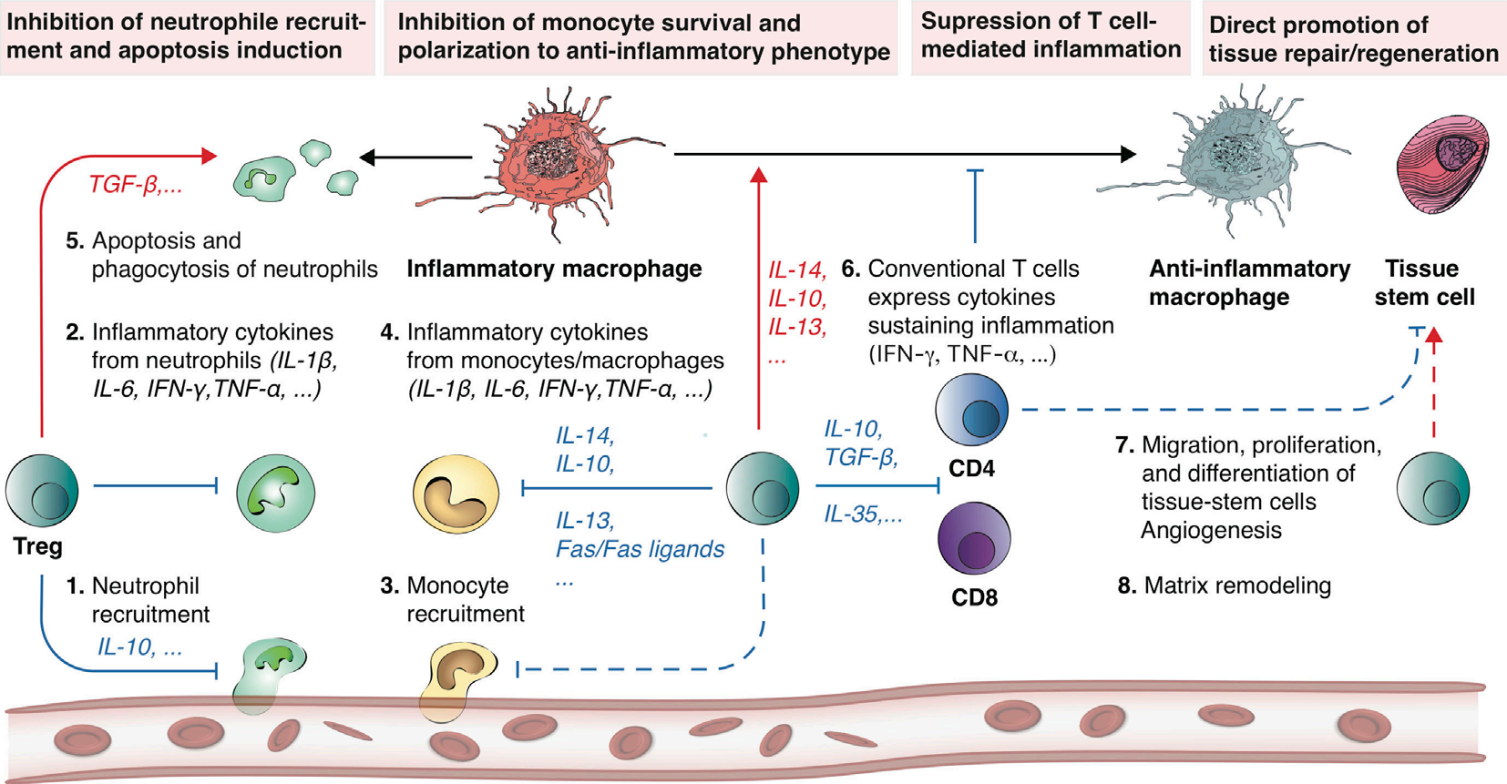
Treg promote tissue repair and regeneration by modulating inflammation. Treg have demonstrated the ability to promote tissue repair and regeneration by controlling both the innate and adaptive immune systems. Following tissue injury, a cascade of immune events is triggered (steps 1–6) until a new tissue is formed (steps 7–8). Treg are involved in all these different steps. At the onset of inflammation, Treg can neutralize inflammatory cytokine secretion (e.g., IL-6, IFN-γ, TNF-α, and IL-1β) by inhibiting neutrophil extravasation *via* IL-10. In addition, Treg are able to promote apoptosis of neutrophils and encourage phagocytosis of dead neutrophils by macrophages. Concomitantly, Treg further inhibit monocyte activity, survival, and stimulate macrophage polarization toward an anti-inflammatory phenotype (M2) *via* the release of anti-inflammatory cytokines (e.g., IL-4, IL-10, IL-13). Similarly, Treg have the natural ability to suppress CD4 and CD8 T cell-mediated inflammation (*via* IL-10, TGF-β, and IL-35). Overall, these Treg-mediated mechanisms result in the inhibition of neutrophil, inflammatory macrophage, as well as CD4 and CD8 T-cell activity, which is generally favorable for tissue repair and regeneration. Dashed lines indicate a hypothetical mechanism. Red arrows indicate induction, while blue arrows indicate inhibition.

Uncontrolled inflammation after tissue injury can lead to impaired healing and tissue remodeling. In many tissues, Treg are recruited to the damaged site to facilitate inflammation resolution and to regulate immunity after injury (19). For instance, Treg can indirectly modulate regeneration by controlling neutrophils (20–22), inducing macrophage polarization (23, 24), and regulating helper T-cells (22, 25) (Figure 1) Moreover, Treg have been shown to directly facilitate regeneration *via* activating progenitor cells locally (16, 17).

## Treg Interact with Innate Immune Cells to Control Inflammation After Tissue Injury

Treg are able to control the functions of neutrophils and macrophages, which have been widely shown to be involved in the tissue healing process. Neutrophils are among the first leukocytes recruited to the injury site, and they directly modulate tissue healing either positively or negatively. For instance, after skeletal muscle injury, it has been demonstrated that neutrophils impair restoration of muscle structures and function through the release of hypochlorous acid, NAPDH oxidase, and other cytokines (26, 27). A negative role of neutrophils has also been demonstrated in a lung ischemia-reperfusion model, where neutrophils enhance the injury (28). However, in an inflammatory lung disease model, mice treated with intratracheal LPS, which induces neutrophil transmigration show activated β-catenin signaling in lung epithelial cells, triggering repair of the lung epithelium (29). Therefore, it is likely that neutrophils modulate tissue healing in a context-specific manner.

Treg have shown ability to modulate tissue healing *via* controlling neutrophil behavior. For instance, an *in vitro* study has demonstrated that activated Treg promote neutrophils to secrete anti-inflammatory molecules including IL-10 and TGF-β, heme oxygenase-1, and indoleamine 2,3-dioxygenase (IDO). This is also preceded by inhibition of neutrophil’s IL-6 production, suggesting that Treg can modulate inflammation through inhibition of neutrophil activity (30). Concomitantly, Treg have been shown to induce neutrophil apoptosis and death both *in vitro* and *in vivo* (21, 31). For example, in an acute lung injury model, Treg mediate resolution of lung injury *via* TGF-β-induced neutrophil apoptosis (21). In addition, Treg can modulate neutrophil infiltration to the site of injury. For instance, deletion of Treg leads to increased infiltration of neutrophils after cardiac injury and subsequently results in impaired healing (20, 22). In a model of kidney ischemia reperfusion injury, Treg suppress infiltration of neutrophils and attenuate kidney injury *via* IL-10 secretion (32). These studies indicate that Treg-mediated modulation of neutrophil behavior and activity is an important step toward regulating tissue healing.

Asides from neutrophils, Treg interact with other key innate immune cells involved in the inflammatory response such as macrophages. In addition to being scavengers that phagocytose cellular debris including apoptotic neutrophils and other cells, macrophages have been shown to play a pivotal role in tissue repair and regeneration. As a remarkable example, salamanders are well-known to be able to regenerate limbs, but depletion of macrophages leads to failure of limb blastemal formation and regeneration (33). Similarly, genetic ablation of macrophages during blastemal proliferation leads to failure of tail fin regeneration in adult zebrafish (34). In mice, depletion of macrophages leads to excessive fibrosis and lack of neoangiogenesis, resulting in failure in neonatal heart regeneration after myocardial infarction (MI) or apex resection (11, 24). Likewise, macrophages are important for cardio protection driven by cardiosphere-derived cells (CDCs), a stem-like population derived from cardiac biopsies *ex vivo*. Indeed, systemic depletion of macrophages with clodronate abolishes CDC-mediated cardioprotection and inhibits their regenerative capability in adult hearts after MI (35).

Importantly, during tissue healing, there are at least two different subsets of monocyte-derived macrophages, namely M1 and M2 macrophages. M1 are pro-inflammatory macrophages usually induced by IFN-γ or TNF-α, while M2 are anti-inflammatory usually induced by IL-4/IL-13 or IL-10. In this context, Treg are important regulator of macrophage phenotypes and functions (36–38). For example, monocytes cocultured with Treg produce decreased level of TNF-α and IL-6 in response to LPS; and the inhibition is associated with secretion of IL-10, IL-4, and IL-13 by Treg (39). Additionally, coculture of monocytes with Treg induces macrophages to polarize toward a M2 phenotype characterized by the upregulation of CD206, CD163, and decreased expression of HLA-DR (40). Treg can attenuate tissue injury and help tissue repair also by modulating macrophage activity and survival. For example, in a chronic kidney disease model, Treg protect kidney injury through inhibition of macrophage activity, which is dependent on Treg-derived TGF-β (41). In this context, Treg also inhibit monocyte survival through the Fas/FasL pathway (41).

## Treg Facilitate Tissue Healing *via* Regulation of Conventional T–Cell Activities

Mounting evidence suggest that conventional T-cells are most likely detrimental for tissue healing (42). For example, CD4- and CD8-deficient mice have improved renal function in renal ischemia reperfusion model. SCID mice, which lack lymphocytes have significantly decreased intestinal leakage of albumin compared to wild-type mice after mesenteric artery ischemia and reperfusion (43, 44). In a MI model, CD8^+^ cytotoxic T-cells can respond to cardiomyocytes after being exposed to autoantigen *in vivo* and kill healthy cardiomyocytes *in vitro* (45, 46). Moreover, *Rag^−/−^* mice, which lack T-cells have significantly smaller infarct size compared to control mice (47). In the context of bone, conventional T-cells may inhibit regeneration by promoting osteoclast differentiation (48). In addition, recruitment of CD8^+^ effector T-cells is correlated with delayed fracture healing and osteogenesis, due to secretion of IFN-γ and TNF-α (49). Deletion of CD8^+^ T-cells in mouse osteotomy model enhances fracture healing while adoptive transfer of CD8^+^ T-cells results in impaired healing (49).

**Figure 2:**
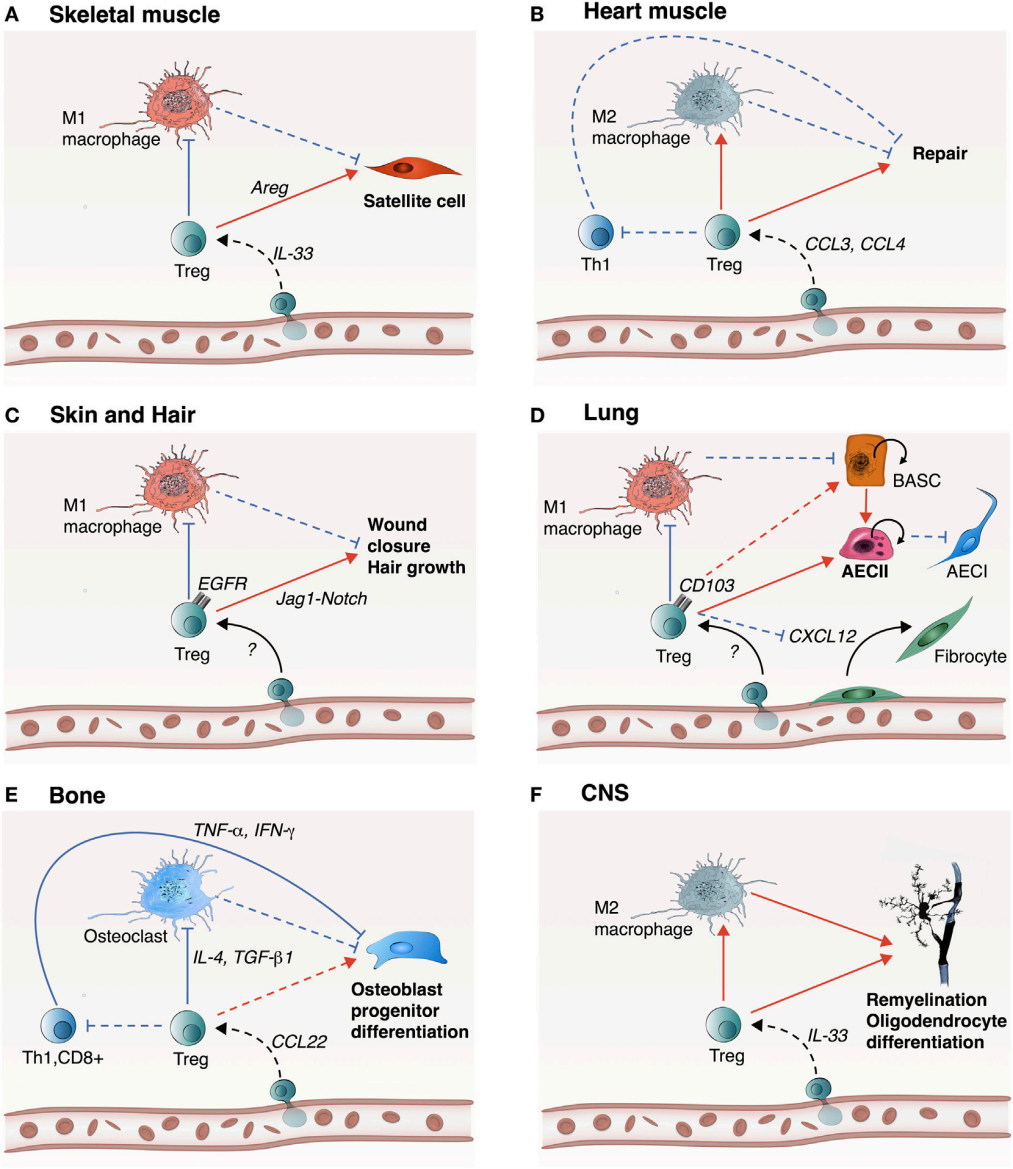
Treg likely promote tissue repair and regeneration in a tissue-specific manner. Treg play an important role in the repair and regeneration of skeletal muscle, heart muscle, skin and hair, lung, bone, and central nervous system (CNS). **(A)** In skeletal muscle, IL-33 participates to Treg recruitment into the site of injury. Treg inhibit M1 macrophage-mediated inflammation, which promote transition to the resolution phase. Treg also directly activate satellite cell proliferation and differentiation through Areg. **(B)** In the heart, Treg are recruited via CCR5 signaling (e.g., CCL3 and CCL4) allowing inhibition of Th1 cell activity and inhibition of M1 macrophages. **(C)** In skin and hair, mechanism of Treg recruitment is still unknown, but upon recruitment, Treg inhibit M1 macrophage inflammatory activity and promote wound closure and hair growth *via* the Jag1-Notch signaling pathway. **(D)** In the lung, Treg inhibit M1 macrophage inflammatory activity and encourage proliferation and differentiation of damaged alveolar type 2 epithelial cells (AECII) into AECIs. This step can be mediated by Areg or CD103 to E-cadherin ligand-receptor binding. Alternatively, Treg could potentially activate progenitor bronchioalveolar stem cells (BASCs) to differentiate into AECII cells. Concurrently, Treg prevent fibrosis by inhibiting fibrocyte recruitment and proliferation *via* CXCL12. **(E)** In the bone, Treg are most likely recruited *via* CCL22, which act on inhibiting Th1, CD8^+^, and M1 macrophages to support osteoblast progenitor differentiation. **(F)** In CNS, Treg are recruited by IL-33 and play a reparative role by encouraging M2 macrophage polarization to facilitate re-myelination and differentiation of oligodendrocytes. Treg may also directly act on oligodendrocytes *via* CCN3. Dashed lines indicate a hypothetical mechanism. Red arrows indicate induction, while blue arrows indicate inhibition.

The negative role of conventional T-cells in tissue injury is most likely mediated by the expression of inflammatory cytokines such as TNF-α and IFN-γ (50–52), but Treg can suppress conventional T-cells through various mechanisms including secretion of anti-inflammatory cytokines such as IL-10, TGF-β, and IL-35 (53–56). For example, it has been shown that deletion of Treg increase CD4^+^ and CD8^+^ cell number in the heart injury zone. In this context, both CD8^+^ and CD4^+^ T-cells show an increased secretion of IFN-γ and TNF-α, suggesting that Treg not only decrease the infiltration of conventional T-cells, but also attenuate their activity (22). Yet, the mechanisms by which Treg modulate tissue repair and regeneration are most likely tissue-dependent. In the next sections, we will discuss the role of Treg in the repair and regeneration of various tissues including skeletal and heart muscle, skin, lung, bone, and the central nervous system (CNS) (Figure 2).

## The Role of Treg in Tissue-Specific Repair and Regeneration

### Skeletal Muscle

It has been shown that Treg accumulate in the skeletal muscle of acutely injured mice or in mdx model of Duchenne muscular dystrophy (18, 57). Normal repair of skeletal muscle is found to require local expansion of Treg, since Treg ablation following treatment with diphtheria toxin in *Foxp3^DTR^* mice or following treatment with the depleting anti-CD25mAb targeting CD4^+^CD25^hi^ Treg increases muscle damage in dystrophic mice (18, 57). Similarly, treatments that enhance Treg activities including complexes of recombinant IL-2 with anti-IL-2 mAb prevented muscle damage in dystrophic mice (18, 57). Comparing the transcriptome of Treg isolated from regenerating muscle and lymphoid tissues including spleen and lymph nodes, Treg from muscle, but not naïve Treg from lymphoid organ express the growth factor Amphiregulin that directly acts on muscle satellite cells *in vitro* and improves muscle repair *in vivo* (18). Depletion of Treg also leads to increased muscle inflammation characterized by an increased IFN-γ response and activation of M1 macrophages (57). Moreover, it has been shown *in vitro* that coculture of induced Treg with muscle satellite cells enhances muscle satellite expansion and inhibits their myogenic differentiation (16). Nevertheless, direct evidence of Treg converting satellite cells into muscle has yet to be demonstrated.

To date, factors, which contribute to the accumulation of Treg in damaged tissues remain elusive. Nevertheless, IL-33 has been shown to facilitate recovery after CNS injury (58) and to drive accumulation of Treg in visceral adipose tissue of lean mice (59) and damaged muscle in young mice (60). IL-33 acts on the suppression of tumorigenicity 2 (ST2) receptor of Treg. Treg devoid of ST2 due to Treg cell-specific ablation of the *Il1rl1* gene show impaired recruitment to injured muscle, resulting in delayed muscle regeneration (60). Moreover, aged mice with more severely impaired muscle repair are found to have less IL-33-dependent accumulation of Treg after acute injury compared to young mice (60). Thus, IL-33 is important in mobilizing Treg in muscle and supplementation of IL-33 can reverse these effects and facilitate muscle regeneration in aged mice (16, 60).

### Heart Muscle

Scarring of cardiac tissue after MI is likely the most deadly injury in humans (61). MI leads to a loss of large number of cardiomyocytes that are unable to regenerate, which ultimately progresses to cardiac failure. Cardiomyocyte death results in replacement by scar tissues and ventricular remodeling that further compromises heart function. Early cardiac wound healing is characterized by infiltration of both innate (62) and adaptive (63) immune cells into the myocardium. In patients with acute MI, increased systemic markers of inflammation correlate with higher mortality (64). Moreover, infiltration of activated CD4^+^CD25^+^ T-cells has been observed in the infarcted and remote regions of myocardium and heart-draining lymph nodes in patients with MI (63, 65). It has also been found that T-cells become activated in patients with coronary artery disease or a history of MI (65, 66). Nevertheless, the role of T-cells during pathogenesis or healing of the human heart is yet to be identified.

In mouse models of CD4^+^ T-cell deficiency, including CD4 or MHC-II knockout mice, or TCR specific for an irrelevant ovalbumin-derived peptide in transgenic OTII mice, CD4^+^ T cell-deficient mice show increased cardiac inflammation, impaired wound healing, left ventricular remodeling, and impaired survival (63). Although myocardial antigens are minimally accessible by the immune system, both *MhcII^−/−^* and OT-II mice have a higher rate of myocardial ruptures and mortality than wild-type mice. This suggest that CD4^+^ T cells are activated after MI driven by recognition of cardiac autoantigens on MHC-II and facilitate healing of the myocardium in an antigen-specific manner (63).

Indeed, T-cells specific for myocardial proteins exist in mice. Both immunization with troponin or myosin-derived peptides, and adoptive transfer of myosin-derived peptides loaded dendritic cells induce myocarditis in susceptible mice (67, 68). CD4^+^ T-cells are also found to be reactive to troponin, a complex comprised of three regulatory proteins troponin-C, -I, and -T, which are integral to cardiac muscle contractility (69). Moreover, the cardiomyocyte-specific protein, α-myosin heavy chain (α-MHC), is not expressed within the thymus of mice and human, and it resembles a non-self protein that activates CD4^+^ T-cells after MI (70). Thus, tolerance to α-MHC reactive T-cells is probably maintained by Treg to prevent autoimmunity after MI. The detrimental role of conventional CD4^+^ T-cells in MI healing most likely involves the adenosine receptor, since adenosine receptor depleted CD4^+^ T-cells are not able to recapitulate the injurious action of CD4^+^ T cells (47). *In vitro* activated Treg cells attenuate myocardial injury through expression of CD39, which promote extracellular degradation of nucleotides to form adenosine (71). Therefore, Treg in heart may function through CD39-mediated adenosine formation.

Treg also improve healing after MI by modulating monocytes and macrophages (22). Treg depletion in *Foxp3^DTR^* mice or following treatment with anti-CD25 mAb show increased infiltration of M1 macrophages, reduced cardiac function, and pronounced left ventricular dilation after MI (22). On the other hand, preferential induction of Treg *via* treatment with superagonistic anti-CD28 mAb leads to increased Treg infiltration into the infarcted myocardium after MI. The higher number of Treg promotes macrophages to polarize toward a M2 phenotype in the healing myocardium and reduce ventricular ruptures, which lead to better survival (22). Mechanistically, CCR5 is associated with Treg recruitment as CCR5 knockout mice show impaired Treg infiltration as well as adverse remodeling and cardiac deterioration after MI (72). Therefore, CCR5-mediated Treg recruitment restrains inflammation, excessive matrix degradation, and adverse remodeling after MI (72).

### Skin and Hair

Layers of murine and human skin contain a large number of resident Treg (73–75). During a short-defined window of postnatal development, Treg migration to neonatal skin is important for the establishment of immune tolerance to commensal microbes (73). Upon entry to the skin through CCL20/CCR6 mediated migration, Treg localize and accumulate in hair follicles (76). Marked accumulation of CD4^+^ T-cells are observed in wounded skin with peaked infiltration at day 7 following injury. Interestingly, the majority of CD4^+^ T-cells are highly activated Treg characterized by increased expression of CD25, CTLA-4, and ICOS (77). Treg depletion following treatment with diphtheria toxin in *Foxp3^DTR^* mice after skin wounding results in significant attenuation of wound closure accompanied by increased tissue granulation and overlying eschar, indicating that Treg facilitate skin wound healing (77). Treg depletion also leads to an increased number of IFN-γ producing T-cells with augmented accumulation of proinflammatory macrophages in wounded skin. Moreover, ablation of epidermal growth factor receptor (EGFR) signaling in Treg using the *Foxp3-Cre*;*EGFR^fl/fl^* mouse model results in reduced Treg infiltration in the wounded skin and significantly delayed wound closure, indicating that the EGFR pathway plays a role in Treg activation and function during skin wound healing (77).

In alopecia areata disease displaying phenotype of hair follicle regeneration, genome-wide association studies have revealed single nucleotide polymorphisms in genes including *Cd25, Ctla4, Eos*, and *Foxp3*, which are important in differentiation and function of Treg (78–80). Treg in skin preferentially localize to hair follicles (81, 82) and are more abundant in the resting telogen than growing anagen phase during hair follicle cycling (17). More importantly, in the telogen phase, Treg display a highly activated phenotype. Transient or constant ablation of Treg in *Foxp3^DTR^* mice following treatment with diphtheria toxin leads to markedly reduced anagen induction in skin and subsequently reduced hair regrowth, indicating the important role of Treg in facilitating hair follicle regeneration by promoting the telogen-to-anagen transition (17). Immunofluorescence microscopy on dorsal skin derived from *Foxp3^GFP^* reporter mice further revealed that Treg preferentially localize to hair follicular stem cell niche, promoting proliferation and differentiation of hair follicular stem cells (17). By comparing the transcriptome of Treg derived from telogen skin and skin-draining lymph nodes, Treg of the skin expressed more *Jag1* lymph node-Treg. Conditional ablation of *Jag1* in Treg of *Foxp3-Cre^+/+^*;*Jag1^fl/fl^* mice significantly attenuates hair follicular stem cell proliferation, suggesting that the Jag1–Notch signaling pathway is essential in facilitating Treg-mediated hair follicle regeneration (17).

### Lung

As microbes and other airborne materials can be frequently aspirated into the lung, pulmonary disease is easy to develop when the pulmonary immunity fails to protect the lungs during infections (83). However, the role of Treg during lung infections has been investigated in mouse models and has resulted in contradictory findings. It has been shown that patients with acute respiratory distress syndrome have increased Treg in their bronchoalveolar lavage fluid, suggesting that Treg play a role in the disease (21). In preclinical models of lung injury, T-cell deficient mice (*Rag-1^−/−^*) showed delayed lung resolution governed by high lung permeability as well as elevated number of neutrophils and macrophages, indicating that T-cells may play a reparative role during lung injury resolution (21). Moreover, further analysis has shown that infiltration of CD4^+^CD25^+^FOXP3^+^ Treg in the alveolar compartment increases upon LPS instillation (21). Furthermore, adoptive transfer of wild-type CD4^+^CD25^+^ splenocytes following intratracheal LPS instillation into *Rag-1^−/−^* mice successfully facilitates lung injury resolution, suggesting that Treg could serve as a rescue therapy after acute lung injury (21). Indeed, Treg play a central role in lung resolution, since adoptive transfer of Treg from wild-type into *Rag-1^−/−^* mice has shown to decrease the number of fibrocytes in LPS-treated lungs. Mechanistically, Treg reduce lung epithelial CXCL12 concentration which is responsible for CXCR4^+^ fibrocyte recruitment (84). In addition, Treg mediate resolution by stifling pro-inflammatory macrophage response and ultimately promote bronchioalveolar stem cells (BASCs) proliferation (84).

The adult lung has a remarkable regenerative potential after injury (85, 86). The alveolar compartment comprises largely (90–95%) of alveolar type I cells (ATI) involved in gas exchange and to a lesser amount (7%) of type II cells (ATII), which are involved in immune regulation, repair, and recovery (87). These cell types derive from BASCs found at the bronchioalveolar duct junction (88). In a left unilateral pneumonectomy mouse model, surgical removal of the left lung induces mass expansion in the intact lobes of the remaining right lung (89). This extravagant alveologenesis process is shown to be dependent on lung epithelial proliferation, specifically through ATII cells responsible for maintaining ATI number through differentiation (89). In acute lung injury or partial pneumonectomy models, it has been shown that epithelial proliferation during lung recovery is significantly impaired after specific elimination of Treg in *Foxp3^DTR^* mice following diphtheria toxin treatment (90). Lung epithelial proliferation is strongly correlated with Treg number after injury and Treg promote ATII proliferation through binding of their surface integrin CD103 to E-Cadherin expressed by epithelial cells (90). Furthermore, the growth factor amphiregulin (Areg) expression by Treg also seems to play a non-redundant role in lung repair (18). Rapid increase in expression level of Areg in lung tissues is observed at day 3 postviral infection. Using *Foxp3-Cre*;*Areg^fl/fl^* mice in which Areg is specifically ablated in Treg, it has been shown that the immunosuppressive function of Treg is preserved during antiviral immune responses. However, in the absence of Areg production by Treg, impaired recovery of lung function has been found in *Foxp3-Cre;Areg^fl/fl^* mice. Interestingly, by coculturing Treg with IL-18 or IL-33, it has been shown that Areg is induced by activation of IL-18R or ST2, instead of being activated through the TCR signaling pathway (91).

### Bone

The adaptive immune system has been shown to play an important role in bone regeneration. Compared to most tissues, bone is capable of healing without scar tissue formation. The homeostasis of bone is mediated mainly by the interaction between osteoblasts which form bone and osteoclasts which resorb bone. Osteoblasts are principally differentiated from progenitors such as mesenchymal stem cells (MSC), while osteoclasts originate from bone marrow-derived monocytes. Interestingly, MSC can induce Treg from naïve T-cells and promote Treg proliferation through HO-1 (92). While CD3^+^ T-cells support peripheral blood mononuclear cell differentiation into osteoclasts *in vitro*, Treg inhibit such a differentiation through paracrine signaling of TGF-β and IL-4 (93, 94). Moreover, the number of Treg in peripheral blood is inversely correlated to serum marker of osteoclastogenesis in normal human and rheumatoid arthritis patients. *In vivo* experiments have also shown that Treg protect TNF-α-induced bone destruction and ovariectomy-induced bone loss (95, 96). Treatment with superagonistic anti-CD28 mAb ameliorates TNF-α-induced arthritis and increases bone mass in wild-type mice. The protective role of Treg in bone loss is most likely the result of impaired osteoclast differentiation and bone resorption *via* inflammatory cytokines. For example, in an actinobacillus actinomycetemcomitans-induced canine model, Treg are recruited to the site of injury by CCL22 and decrease bone resorption through reducing inflammation (97). Interestingly, Treg may also directly promote osteoblast differentiation from progenitor cells. For instance, it has been demonstrated that Treg facilitate MSC-based bone regeneration by inhibiting CD4^+^ conventional T-cells, which secrete IFN-γ and TNF-α (52, 98).

### Central Nervous System

In mice deficient for CD4^+^ or CD8^+^ T-cells, remyelination is inhibited after lysolecithin injection, suggesting that CD4^+^ and CD8^+^ T-cells are required in remyelination of the CNS (99). In a myelin oligodendrocyte glycoprotein-induced experimental autoimmune encephalomyelitis mouse model, Treg are found to expand in peripheral lymphoid compartment and accumulate in CNS (100). Even though infiltrating Treg fail to control autoimmune inflammation, it has been demonstrated that they promote myelin regeneration (25). Moreover, Treg-deficient mice show impaired remyelinataion and oligodendrocyte differentiation that can be rescued by adoptive transfer of Treg. IL-33 has been found to promote Treg recruitment into injured tissues, facilitating recovery after CNS injury. In addition, mice lacking IL-33 have impaired recovery after CNS injury, which is associated with reduced myeloid cell infiltrates and decreased induction of M2-assiociated genes at the injury site (58). Treg also promote oligodendrocyte progenitor cell differentiation and myelination *in vitro* and *ex vivo*. Interestingly, through proteome profiling of Treg conditioned media, nephroblastoma overexpressed, also known as CCN3, has been found to mediate Treg-driven oligodendrocyte progenitor cell differentiation and CNS myelination (25).

## Future Perspectives

Recently, exploring the function of the immune system during tissue repair and regeneration has gained a lot of interest in regenerative medicine. Nevertheless, we still have sparse knowledge on how immunity—in particular the adaptive immune system—controls the tissue healing process. For instances, what are the neo antigens, if any, released to initiate adaptive immunity-mediated tissue healing? Similarly, is adaptive immunity-mediated tissue healing an antigen-specific process? If so, how are T-cells recruited, activated and function in response to self-antigens during injury that are different from responding to non-self antigens? Currently, the development of treatments targeting the immune system is hindered by the lack of markers that specifically define distinct subsets of immune cells. Recent advances in single-cell genomics could offer unprecedented delineation of lineage-specific markers and function of various subsets of immune cells operating during tissue repair and regeneration.

Furthermore, it is still unclear why scars are absent in some tissues such as in bone, but are forming in others such as in heart. Understanding how both innate and adaptive immune cells interact with tissue-resident progenitor cells and myofibroblasts would shed light on developing therapeutic strategies for improving healing and regeneration in the clinic. Moreover, accumulating evidence has shown that the function of the immune system declines with age (101, 102). Given that the immune system plays a crucial role in tissue repair and regeneration, whether the reduced tissue repair capacity is related to a degenerated immune system during aging awaits further investigations.

Overall, studies investigating the role of Treg during tissue regeneration have been largely based on the use of *Foxp3^DTR^* mouse model (17, 18, 22, 25, 57, 77, 90). However, one caveat of using such model is that the mice develop spontaneous systemic autoimmunity when Treg are depleted for long term (103). Careful data analysis should also include gain-of-function experiments such as adoptive transfer of purified Treg into *Rag1^−/−^* mice to determine the role of Treg in tissue repair and regeneration.

From a regenerative point of view, one could control tissue Treg to promote regeneration. Treg of adipose tissues, skeletal muscle, and colonic lamina propria are the best characterized tissue Treg that maintain organismal homeostasis (104). Similar to regenerative Treg as aforementioned, IL-33 has been reported to expand tissue Treg in colonic lamina propria (105) that are well equipped to participate in local repair responses with expression of the tissue repair factor, amphiregulin. The exact role of these tissue Treg in intestinal regeneration awaits further investigations. Nevertheless, manipulating Treg for alleviating inflammatory diseases has been tested in several clinical trials. For instances, the use of low dosage of IL-2 in selective expansion of Treg in human patients (106) as well as *ex vivo* expansion and adoptive transfer of Treg to treat Type 1 diabetes have been reported and tested in the clinic (107–109). Further studies are needed to investigate if these Treg-mediated strategies can also be utilized for inducing tissue regeneration.

Although it has been reported that superagonistic anti-CD28 mAb increases Treg infiltration or activities in mice, the use of humanized superagonisticanti-CD28 antibody TGF1412 caused cytokine storm, leading to organ failure in a previous trial (110). Furthermore, even though IL-33 plays an important role in recruitment and function of Treg in mice (60), IL-33 is dispensable in humans as individuals lacking IL-33 have no obvious health problems such as autoimmunity, indicating that the pro-regenerative function of IL-33 on Treg could also be different between mice and humans (111). Therefore, novel strategies in empowering Treg-mediated tissue regeneration for potential clinical uses would be needed in the future.

## Author Contributions

JL and KL wrote the manuscript; JT and MM made the figures; MM and KL revised the manuscript.

## Conflict of Interest Statement

The authors declare that the research was conducted in the absence of any commercial or financial relationships that could be construed as a potential conflict of interest. The reviewer AA and handling Editor declared their shared affiliation.
